# A Consideration of the Anthrax Inoculations of Pasteur*Translated from the Vorträge für Thierärzte. 7th Series, No. 1.

**Published:** 1885-01

**Authors:** H. Putz


### ART VIII.—A CONSIDERATION OF THE ANTHRAX INOCULATIONS OF PASTEUR.*

BY PROF. H. PUTZ.

In the year 1879 Pasteur communicated to the Adademy of Sciences in Paris that he had succeeded in cultivating the Virus of Hen Cholera in such a manner as to greatly reduce its virulence so that it could be safely inoculated on fowls, and that, when thus treated, they were rendered unsusceptible to natural infection.

Six months later, Toussaint, Professor at the Veterinary School in Toulouse, France, also sent a communication to the Academy that he was able to produce immunity against anthrax in animals by means of a cultivated and diluted preparation of the virus. The communication was, however, of a private nature. This raised discussion, and caused Toussaint to make public the peculiarities of his method. He authorized Bouley to open the document, and publish its contents. The essentials of his method were, that he inoculated animals with blood serum taken from known anthrax cases which had been subjected to especial treatment in order to mitigate its virulency. This end was attained in two different ways:

* Translated from the Vorträge für Thierärzte. 7th Series, No. 1.

First. The blood from animals diseased with anthrax was defibrinated and subjected to the influence of a temperature of 55° C. for about ten minutes, and after being cooled, used as a fluid for inoculation.

Second. He treated such blood with carbolic acid—manner not given—and used it for inoculations.

Toussaint assumed that the first method of treatment caused the death of the anthrax-bacilli without injuring the infectious qualities of the material, but at the same time it was deprived of a portion of its virulence. He soon acknowledged that he was in error, however.

Lœffler has shown that subjecting anthrax blood to 55° C. for twenty minutes is not sufficient to deprive it of its virulent properties, *i. e.*, to kill the bacteria, but that it requires thirty minutes to bring out such a condition. Inoculations with such material are always followed by negative results, which is not the case with Toussaint's preparation, by which the virulence is not taken from the material, though somewhat lessened in degree.

Lœffler has further shown that the addition of carbolic acid to a medium containing anthrax bacteria, when it amounted to 1 to 1½ per cent., destroyed its virulency, but that ¼ per cent. did not. Blood containing anthrax bacilli is borne by many animals when mixed with a 1 per cent. carbolic acid solution in equal parts. According to Toussaint, it is this mixture that has been used in his experiments. A solution of ½ per cent. carbolic acid does not remove the virulence from anthrax material. Toussaint has made quite a number of experiments upon sheep with material prepared in the above given manner, and assumes that he has discovered that it protects these animals from natural infection by the elements of anthrax. In consequence of these communications Bouley induced the French Ministers of Agriculture to place quite a number of sheep at the disposal of the Alfort Veterinary School, in order to decide this question, which is of immense national importance.

On the 26th of August, 1880, Toussaint began the inoculation of twenty sheep which were placed under the control of the faculty at Alfort. Bouley reported upon the results of these experiments at a meeting of the Academy held in Paris on the 21st of September, 1880. Toussaint brought with him to Alfort, two vials containing fluid for the inoculation, that had been prepared in Toulouse some ten days previously. The sheep at Alfort were inoculated with a proportionally small amount of the same. Within the first four days subsequent to the inoculation four sheep died, and as their blood was found to be filled with anthrax bacilli there could be no doubts raised as to the *causa lethalis.* The remainder were sick, some severely, but all finally recovered. These were afterwards inoculated with pure anthrax material, and it was proven that the sixteen remaining sheep had acquired immunity against further infection. Toussaint had obtained similar results in eleven sheep treated in the same manner at Toulouse. Although these experimental inoculations were not found to be without some danger to the animals, still they conclusively proved that anthrax could be classed with those diseases which produced a certain degree of immunity in some of our domestic animals, viz: sheep, after one attack. The duration of this period of immunity could not be at present decided upon.

Toussaint warns against repeating the inoculation at too short an interval after the primary has taken place, and places the time at about twelve days subsequent, as he has learned by experience that cumulative action may be expected if the secondary or controling experiment is resorted to earlier. The *centrale* inoculation should only be made when all indications of the primary have disappeared. Chauveau has also recorded experiences confirmatory of the above. He also discovered that Algerian sheep possessed but little susceptivity to anthrax infection, for when he inoculated such with a moderate quantity of undiluted anthrax virus, they did not die, although symptoms of the disease could be distinguished in them. Secondary inoculations still more increased the immunity of these sheep towards anthrax contagion, if sufficient time was allowed to elapse (twelve days), but if not they caused death.

In No. 13 of the "Sammburg Klinischer Vorträge, Volkmann, 1882," is an interesting article from Eberth, which contains many valuable communications, as also some of a questionable nature. He remarks that anthrax can only be generated by specific bacilli, but not to the same degree in all animals, as these bacteria are remarkably sensitive to the nature of the nourishment offered by the constitution of the different animals, rats possessing a varying degree of immunity towards infections, while mice are very susceptible. Pasteur has not hesitated in pronouncing the results of his experiments on sheep as being somewhat questionable as to cattle, and has retained his affirmation of the preventive power of anthrax inoculation only for those animals by which he has obtained directly positive results.

Pasteur made his first communication to the Academy upon this subject on the 28th of February, 1881, in which he asserts that he has succeeded in preparing a lymph-fluid, by means of the artificial cultivation of the bacteria of anthrax, which can be used without danger to the animals for the prevention of anthrax. He can prepare this fluid in any degree of dilution that he desires by cultivating anthrax bacilli in bouillon made from chicken meat, in a temperature of 42 deg. to 43 deg. C., and allowing the free access of filtrated air. The infectiousness diminishes, with the prolonged action of oxygen, in such cultivations. His inoculation experiments with dilute cultivated virus made upon rabbits, guinea-pigs, and sheep, gave the most satisfactory results. The animals became only slightly, and in many cases were not perceptibly ill, and were immunable to any action of even natural virus after the results of the second inoculation had disappeared.

These experiments led the Agricultural Society at Melun to invite Pasteur to conduct a series of experiments under their auspices, which demonstrates the importance which these researches had assumed in the minds of the agricultural leaders of France. The Society placed sixty sheep and ten head of cattle at Pasteur's disposal. He promised, however, successful results with regard to the sheep only. The first inoculation occurred on the 5th of May, 1881; the second on the 16th, with the cultivated lymph; on the 31st the *centrale* inoculations were effected on twenty-five sheep, in concurrence with the same on twenty-five that had not been thus treated with genuine anthrax material. On the 2d of June twenty-four of the sheep that had not been through the preventive treatment were dead, and the twenty-fifth dying; while those that had been treated with the cultivated inoculations remained perfectly well, scarcely a rise in temperature being perceptible. Subsequent comparison of these twenty-five sheep with ten others that had been reserved for still further *centrale* experimentation, proved that the health of the former had been in no way interfered with.

The results of these experiments have infinitely more weight than all the opposition which soon followed to Pasteur's experiments. Pasteur also treated some 200 sheep at a farm near Vincennes belonging to the Alfort Veterinary School. I myself saw these sheep in August, 1881, and could observe nothing abnormal in them, save two holes in the ear where the preventive inoculations had been made. Two of these sheep were removed to Alfort after time enough had elapsed for them to acquire immunity, and with two that had not been so treated, were inoculated with a cultivated well known deadly material before the students. The two former sheep remained perfectly well, while both of the others died of anthrax on the second subsequent day.

These favorable results caused an immense degree of excitement in medical and agricultural circles all over Europe. It was doubted if such sheep could withstand natural infection, even though they possessed immunity against a cultivated virus of known deadly quality.

This question was taken up by a commission composed of veterinarians, doctors and agriculturists. On the 16th of July, 1881, they took 16 of the preparatory inoculated sheep from the farm at Vincennes, and 16 from Beauce, that had not been so prepared, and inoculated them with fresh material, in large quantity, taken from a sheep that had died from natural anthrax but four hours previously.

Three of the sixteen non-prepared sheep died of anthrax on the 17th of July; ten on the 18th, and three more on the 19th, while the prepared sheep from Vincennes all remained healthy, although each received, as did the others, 5 ccms. of the blood of the sheep that died of natural anthrax.

Although many skeptics were present at these experiments, the most doubtful of them became convinced of the value of Pasteur's procedure. The negative and in part doubtful results obtained by Oemler and Löffler are completely neutralized by such brilliant success as the above.

Incited by the above, the Hungarian Department of Agriculture invited Pasteur to demonstrate his inoculative treatment in their country. He was unable to accept the offer in person, but detailed an assistant, Thuillier, to visit Budapest—fall of 1881—and meet the wishes of the ministers. A commission of veterinarians, doctors, and others interested, was appointed. The results were published by Dr. Roxahiggi in the Deutschen Medical Wochenschrift, January 7, 1882, from which we take the following:

Anthrax bacilli develop most favorably in meat-bouillon in a temperature of from 25 to 40° C.; when the temperature is of a higher or lower degree the development decreases and ceases entirely by 15° C. or 45° C. Supported by the fact that birds, especially carnivorous ones, promise a certain degree of immunity against anthrax, Pasteur decided to cultivate blood containing anthrax bacilli in bouillon at a temperature of 42 or 43° C. He found that the bacilli grew to long threads, but did not develop permanent spores. (It should be remarked that the bacilli of anthrax have the staff and coccus form; the latter being their permanent form, in which they can remain for a long time resistant to many adverse influences). The longer he continued the cultivation under the constant influence of filtrated atmospheric air, the more did the fluid lessen in virulency, so that after the lapse of 24 hours so much of it had been taken away that sheep could be inoculated without subjecting them to any danger. Inoculations made with this material, *when* 12 *days old*, caused one-half of the animals to die of anthrax.

The bacteria remain active in such cultivations for from 4 to 6 weeks: after this time they gradually die. Wadding in the tube used to convey the atmosphere to the cultivation, answers every purpose of filtration.

The recent endeavor of Pasteur was to produce an inoculation lymph possessing more durable qualities.

Take a drop of the previously mentioned cultivated fluid at any time during the first six weeks after its preparation, and place it in a sterilized (freshly cooked) bouillon, which is to be placed in the cultivating machine at a temperature of 35 deg. C. The bacilli develop very rapidly. This new generation possesses the same degree of virulence that the original did.

After forty-eight hours' exposure to the above-mentioned temperature, the bacteria begin to develop permanent spores, the entire mass assuming this condition in the course of a few days. This *spore-fluid* has the same degree of virulence which the second cultivation had when introduced into the bouillon.

In this way we can produce a fluid of any wished-for value, and which will retain its active properties, if properly closed in tubes, for a long time.

Pasteur designates his twenty-four-days-old culture, as well as his second cultivation, primary vaccine, because he uses it for his first preventive inoculations. This is proportionally without danger to guinea pigs, rabbits, and sheep, as well as the larger domestic animals, while it is still able to kill mice. The twelve-days-old culture and its secondary product Pasteur designates as his second vaccine, while he uses it after the lapse of twelve days from the first inoculation. Twelve days after this second inoculation the animals should, as a rule, have acquired immunity against anthrax infection. At the Veterinary School in Budapest, Thuillier experimented upon six grown cattle and four calves and sixty sheep. The lymph, brought from Pasteur's laboratory, contained spores. Four sheep were directly inoculated with the same, while twenty-six sheep and five cattle were inoculated with freshly cultivated material prepared *en loci* by Thuillier for the occasion. Half of the animals were uninoculated. The first preventive inoculation occurred on the 23d of September, 1881, with primary vaccine ; the second on the 5th of October. On the 8th of October one of these animals died of anthrax ; the others were apparently but little disturbed. On the 17th of October the *centrale* experiments were made upon twenty-five prepared and the same number of non-vaccinated sheep, and five each of similarly treated cattle.

Of the non-vaccinated sheep, the first death occurred within thirty-six hours, and within eight days twenty-three of these animals had died, twenty-two of them surely of anthrax. Of the cattle none died, although one calf was quite sick, but recovered. The vaccinated sheep retained their immunity.

Resumé of the various experiments that have been made under Pasteur's control with his inoculative fluids :

1. The experiments conducted by Pasteur in person have shown that preventive inoculations against anthrax have been made in different places in France upon cattle and sheep without danger.

2. The protective inoculations in France on sheep have proved that such sheep acquire immunity from anthrax. By cattle the results are still uncertain. It is, however, probable, that immunity can also be produced in cattle and other domestic animals.

3. The duration of this immunity has been proven to continue for about a year; it may yet prove to be during life.

4. The experiments made in other places by Pasteur's assistants have not been followed by the same uniformity in results as those done under his personal supervision in France, though by no means undermining the value of his researches.

5. Cattle are less susceptible to inoculative anthrax than sheep.

This subject may well be concluded by Frank's experiment upon the same subject, of which the following are the essentials: *

In the year 1882 the inoculation procedures of Pasteur with reference to anthrax caused a great deal of excitement in scientific circles, the Munich Veterinary School being no exception in this regard.

Anthrax appears most frequently in cattle among the various domestic animals of Bavaria; although sporadic cases continually occur, it is only at intervals that this disease assumes an enzoötic character. These outbreaks take place most frequently in that portion of Bavaria known as the Bavarian Alps. It is an interesting fact that the disease attacks cattle principally, and next in frequency the horse, while, although sheep are much more susceptible to infection, still only isolated cases occur among them. Nevertheless, in the well-known anthrax districts of Upper Bavaria both sheep and cattle are pastured upon the same mountains. This phenomena, which at first appears so singular, finds its logical explanation when we remember that these mountainous pastures are very extensive, and that the sheep seem to have an especial preference for the loftiest portions of the district, while the cattle prefer the more level and better grassed parts of the pastures. It thus

* Jahreschericht d. K. Central Veterinair Schule, Munich, 1882, 1883.

results that while both sheep and cattle may graze upon the same mountain, still their grazing grounds are seldom the same. Where one has opportunity to study the outbreaks of anthrax in these districts he will soon become convinced that only certain localities are characterized by such eruptions, and conceive how it becomes possible for the sheep to remain free while the cattle become a prey to the disease.

These conditions gave us the reason for conducting our inoculation among cattle only. The question for us to decide was:

Does the inoculative treatment of cattle, according to the method of Pasteur, produce in cattle immunity against infection from anthrax? These experiments were made at the Government Station at Leuggries, in Upper Bavaria, a locality where anthrax frequently appears. For this purpose we procured six small cattle, one and a half years old. Other animals were also obtained during the course of the experiments.

The material for inoculation was procured from Boutroux, in Paris, consisting of Pasteur's primary and secondary vaccine. In the same—the examination was only made after the inoculations had been completed—one could see numerous groups of spores, but none of the specific bacilli of anthrax, and a few isolated drops of fatty material. The material was entirely free from any tendency to putrefactive changes, and no other germoid elements could be seen.

On the 5th of August, 1882, we inoculated six cattle with the primary vaccine, a little behind the left shoulder, following Pasteur's instruction to the letter. Each one received $1\frac{1}{2}$ grammes of the material subcutaneously. The temperature of the animals was taken at 7 A. M. and 5 P. M. daily, but no change of note was perceived. Tumefaction did not occur at the point of inoculation.

After the elapse of fourteen days, the 19th of August, the second inoculation with the stronger secondary vaccine of Pasteur occurred, each animal receiving the same amount of this material as they had received in the first instance. We could perceive no reaction following this treatment.

The *centrale* inoculations with genuine material were made upon the 3d and 4th of September, *i. e.*, after the elapse of another fourteen days. To this end a sheep had already been inoculated on the 1st with well known active material; the animal died on the 3d under the characteristic symptoms. For the purpose of these *centrale* experiments, threads were soaked in the warm blood from the jugular of this sheep, and then drawn through the subcutaneous tissues of the left ear for a distance of about 1½ cms. This procedure was considered to promise better results than the subcutaneous introduction of a few drops of blood. On the 3d of September immediately following the death of the sheep, we inoculated No. 4 of the cattle that had been prepared as previously described, as well as a newly purchased non-inoculated one. On the morning of the 4th the remainder of the preventive inoculated cattle, 1, 2, 3, 5, 6, as well as a non-anticipatory-treated steer, No. 8, were also subjected to the *centrale* treatment. No. 4 (prevention inoculated) and No. 7 (not so treated) were also subjected to the action of a piece of material containing anthrax spores, prepared according to Buchner's method, viz: the spores are mixed with a mass made of gum arabic and sugar of milk, and allowed to dry. This was introduced under the skin of the left ear. All of the animals became sick in the same manner, whether they had been subjected to the preventive treatment or not.

In all of them tumefaction occurred at the *locus inoculationis*, being more marked in some than in others; 1, 4, 5, and 7 showed marked swelling, while it was weak in 2, 3, and 6, and moderately prominent in No. 8. Nos. 4 and 7 were notably sick on the 4th of September. On the 5th they were still much depressed, as well as Nos. 3 and 5. The temperature increased in all of them. On the 6th they all had some appetite, though it was weak in Nos. 4 and 7. No. 6 appeared entirely well again. On the night of the 6th of September the non-preventive inoculated animal No. 8 died; and on the 6th, No. 4, that had been subjected to this treatment. With regard to the symptoms, it should be again remarked that all of the animals sickened and two died when inoculated with recognized virulent material. It is not always those animals by which we observe the highest temperature, that die or are always the sickest.

Were one to see such animals upon the pasture and not know that they were infected with anthrax, he would consider them to be perfectly well, unless a special examination were made. The same thing occurs in eruptions of anthrax among the cattle on the mountains. It is probable that in such cases many more cattle have the disease than are suspected by the observer, as all animals complicated by anthrax do not necessarily die, and as we have shown the majority recover even when inoculated with pure anthrax blood. It is therefore highly probable that in the natural outbreaks of anthrax among cattle, many cattle recover without any one having an intimation that the disease has been at work in their systems. This is especially true of young cattle and oxen, while in cows the rapid decrease in the milk production would naturally call attention to the prevalence of disease and the necessity of an exact examination. The circumstance that cattle can thus be attacked by natural anthrax and still recover, is of the greatest practical importance, and seems to have, in general, escaped the observation of veterinarians. The continued study of the inoculation question will show that cattle acquire a more or less extended immunity from anthrax after once having been through with that disease. Accordingly, cattle that have had it in this "still," unperceived manner, upon their grazing grounds, may also possess this acquired immunity. It is probable that many apparently healthy animals would thus appear as if they had been subjected to preventive inoculation after a herd had been through an enzoötic outbreak. This may also be the reason why such eruptions among our Alpine cattle are generally of such short duration, and that in the next, or for several years, eruptions of a like severity do not occur.

This is a point which should require the most exact study in coming years.

Another interesting point is, that while all the inoculated animals had more or less tumufaction at the point of insertion, those that were subjected to feeding experiments with pure anthrax material, did not present such swellings. It is well known that in natural infection many animals have such swellings—of them it has been assumed that many recover—which is not the case in others. The last form is considered the most fatal. It is therefore highly probable that genuine anthrax fever develops when the spores gain access to the organism by means of the digestive tract, while tumefactions result from the outward inoculation by means of worms, flies, etc.

These are, of course, the two ways in which the germs, in general, gain access to the organism. The above-described experimental observations show to a certainty that Pasteur's method of preventive inoculation is not capable of preventing a secondary attack in cattle. As the material gave no indications of having been spoiled, we must conclude that it was too weak to produce this result.

When we pass in review the various experiments which have been made with this material in France and elsewhere, both upon cattle and sheep, we must conclude that much is still wanting to the completion of this method.

In saying this we have no intention whatever of detracting an iota from the true value of Pasteur's discovery. On the contrary, I hold it still to be one of the greatest discoveries of the Nineteenth Century, as the focus of the most important step in experimental prevention of many diseases which will sooner or later be of the utmost practical importance.

The objections to be overcome are: The material of Pasteur is at present very variable in its action—now too weak and again too strong. Hence, for the practical execution of preventive inoculation it would be necessary to first prove the material, which, on account of its fluid form, is at present impossible, as the material once opened to the air is liable to pollution and the consequential change in its nature. A material must be prepared in the future which will admit of such tests without causing injury to it.

It has been shown that Pasteur's material occasionally suffers changes that have been followed by septicæmia, etc., when used for preventive inoculation.

Again, different grades of this material must be prepared and adapted to the constitutional peculiarities of our different animals. In order to arrive at this necessary point the material must be prepared in such a form that it can be used for experiment without the danger of its becoming polluted or injured by contact with air.

Still further feeding and inoculative experiments were made upon those cattle that had withstood the inoculation with genuine anthrax material, and in no case was it possible to produce anthrax in them, even when some six months had elapsed, and the feeding experiments continued for a long time with well known malignant material.

These experiments will be continued in the hope that such graded material may be found, and a manner of preparing it suitable for use at all times with conformable uniformity in results.

The above experiments conclusively show that cattle acquire immunity from second attacks of anthrax if they have once had that disease, and that this immunity exists for some time. Pasteur and others have conclusively shown the value of his preventive treatment for sheep with reference to this same disease.

